# Mapping of a QTL associated with sucrose content in peanut kernels using BSA-seq

**DOI:** 10.3389/fgene.2022.1089389

**Published:** 2023-01-04

**Authors:** Junjia Guo, Feiyan Qi, Li Qin, Maoning Zhang, Ziqi Sun, Hongyan Li, Mengjie Cui, Mengyuan Zhang, Chenyu Li, Xiaona Li, Qi Zhao, Dandan Luo, Mengdi Tian, Hua Liu, Jing Xu, Lijuan Miao, Bingyan Huang, Wenzhao Dong, Suoyi Han, Xinyou Zhang

**Affiliations:** Henan Academy of Crops Molecular Breeding/Postgraduate T&R Base of Zhengzhou University/The Shennong Laboratory/Key Laboratory of Oil Crops in Huang-Huai-Hai Plains, Ministry of Agriculture/Henan Provincial Key Laboratory for Oil Crop Improvement/National Centre for Plant Breeding, Xinxiang, Henan, China

**Keywords:** peanut, sucrose content, BSA-seq, QTL, KASP

## Abstract

As an important factor affecting the edible quality of peanut kernels, sucrose content is a complex quantitative trait regulated by multiple factors. In this study, an F_2_ segregating population and a recombinant inbred line (RIL) population, derived from a cross between the high sucrose content variety Jihuatian 1 and the low sucrose content line PI478819, were used as materials to map a quantitative trait locus (QTL) associated with sucrose content in peanut kernels. Four QTLs were initially located on chromosomes A03 and A06 based on BSA-seq technology, and multiple kompetitive allele-specific PCR markers were developed based on single-nucleotide polymorphisms (SNPs) in the intervals. The markers were genotyped in the RIL population and finely mapped to a stable QTL, *qSUCA06*, located on chromosome A06 within a 0.29-Mb physical genomic interval (112367085–112662675 bp), which accounted for 31.95%–41.05% of the phenotypic variance explained. SNP and insertion/deletion annotations were performed on genes in the candidate interval, and having screened out those genes with mutations in exons, candidate genes were verified by qRT-PCR. The results revealed that *Arahy.Y2LWD9* may be the main gene regulating sucrose content. The QTL identified in this study will not only contribute to marker-assisted breeding for improvement of peanut sucrose content but also paves the way for identifying gene function.

## Introduction

Peanuts (*Arachis hypogaea* L.), an important oil and cash crop, are rich in vegetable oil and protein, and widely cultivated worldwide (Liu et al., 2020a). In recent years, during which there have been increases in the production and consumption of edible peanuts, increasing attention has focused on the edible quality of peanut kernels, an important index of which is sweetness. Indeed, some studies have reported correlation values of as high as 0.88 between sweetness and peanut kernel taste quality (Pattee et al., 1998). The most direct factor affecting the sweetness of peanut is the content of soluble sugars in kernels. These sugars consist primarily of sucrose, fructose, and glucose, among which, sucrose accounts for the largest proportion, and makes the largest contribution to the sweetness of peanuts (McDaniel et al., 2012). Given that peanuts with kernel sucrose contents exceeding 6% are considered to have a better taste (P), determining the main genetic loci controlling sucrose content in kernels would make a valuable contribution to enhancing the sucrose content and edible quality of peanuts.

In plants, sucrose, the main product of leaf photosynthesis, is exported to different non-photosynthetic organs according to demands for the synthesis of carbon and storage materials required for growth (Davis and Dean, 2016). Sucrose transported to the developing seeds is synthesized to yield lipid (oil) or protein storage substances under the action of a series of enzymes such as invertase. Genetic studies have shown that there are multiple factors affecting the sucrose content in kernels, including the influences of environmental factors and plant genotype (Pattee et al., 1981), maturity (Sanders and Bett, 1995), and genotype–environment interactions (Pattee et al., 2000). Furthermore, it has been established that there is a significant difference in the kernel sucrose contents of plants derived from direct and reciprocal crosses, which tends to indicate that this trait is matrilineally determined (Isleib et al., 2004). Collectively, the aforementioned findings provide evidence to indicate that the sucrose content of peanut kernels is a complex quantitative character influenced by multiple factors.

Bulked-segregant analysis (BSA) can be applied to rapidly and efficiently mine causal genes without the necessity of constructing a genetic map. The technique, which uses amplified fragment length polymorphic (AFLP) and restriction fragment length polymorphic (RFLP) markers, was initially used in lettuce and tomato (Giovannoni et al., 1991; Michelmore et al., 1991). The principle of the BSA-seq method is based on the selection of individuals in a population with bipolar characteristics to construct mixed pools, the whole genomes of which are sequenced to identify causal genes associated with traits of interest (Liu et al., 2020b). With the emergence and development of high-throughput sequencing technology, given its high efficiency, the BSA-seq method has been widely used in the analysis of important agronomic characters of soybean (Xie et al., 2021), rice (Yang et al., 2021), sesame (Sheng et al., 2021), and other crops. In peanut, this method has been used for quantitative trait gene mining for traits such as fresh seed dormancy (Kumar et al., 2020), seed coat color (Chen et al., 2021), and late leaf spot resistance (Clevenger et al., 2018; Han et al., 2022).

In this study, using peanut genotypes Jihuatian one and PI478819 as parental plants, we used a combination of BSA-seq and kompetitive allele-specific PCR (KASP) markers to determine the quantitative trait locus (QTL) controlling the sucrose content of peanut kernels and to predict candidate genes. Our findings will provide a theoretical basis for further elucidation of the control of sucrose content in peanuts, and thereby contribute to breeding for enhanced edible quality.

## Materials and methods

### Plant materials and phenotypic evaluation

In the present study, we used the peanut genotypes Jihuatian 1 and PI478819. The high-sucrose variety Jihuatian 1 is a Spanish-type cultivar developed by the Hebei Academy of Agriculture and Forestry Sciences, China, whereas the low-sucrose line PI478819 is a Virginia-type variety introduced from the United States. These germplasms were used as the female and male parents, respectively, which were crossed to obtain an F_1_ population. KASP molecular marker technique was used to identify true and false hybrids of F_1_ seeds. The KASP markers with obvious differences between parents were designed, and the genomic DNA of parents and F_1_ seeds were extracted and detected by KASP molecular markers. The homozygous type with the same genotype as the parent was false hybrid, and the heterozygous genotype was expressed as true hybrid (Qin et al., 2020). A subsequent F_2_ segregating population consisting of 831 lines was obtained by selfing. In addition, a population of recombinant inbred lines (RILs) was obtained based on single-seed descent. F_2_ population and parental individuals were planted on the experimental farm of Henan Academy of Agricultural Sciences in Xinxiang (Henan province) in May 2017, and a total of 251 lines of the RIL population were planted in Xinxiang (Henan), Kaifeng (Henan), and Zhumadian (Henan) in May 2021. Seeds of both the F_2_ and RIL populations were planted individually in holes. For the RIL population, RILs were planted in a randomized complete block design with two replications. Each RIL comprising 10 plants in one replicate was planted in a single row with inter-plant spacing of 0.2 m and inter-row spacing of 0.5 m in each of the testing environments. Crop management was conducted following regular agricultural practices (Zhang et al., 2022).

Mature pods harvested from the experimental plants were naturally sun-dried, and the sucrose contents of peanut kernels were measured using a near infra-red (NIR) spectrometer (DA7200; Perten). For measurement, we selected three replicates of approximately 20 uniform kernels, which were evenly packed into a sample cup, and NIR spectral information was collected in the wavelength range 950–1,650 nm (Qin et al., 2016).

### Mixed pool construction and whole-genome resequencing

Young leaves were collected from all F_2_ population lines and the parent plants, from which genomic DNAs were extracted using a plant genomic DNA extraction kit (DP305-03; TianGen), followed by determination of DNA quality. On the basis of the determined sucrose contents of F_2_ individuals, we selected 20 plants with extremely high sucrose content and 20 with low sucrose content, the respective DNAs of which were mixed in equal quantities to give two extreme phenotypic mixed pools. DNAs from these two mixed pools and both parents were then subjected to whole-genome resequencing using the Illumina HiSeq/DNBSEQ platform in conjunction with a double-terminal 150-bp sequencing strategy. The sequencing depth was 20×, and the reference genome used was the Tifrunner_V20190521 version of cultivated peanut (https://www.peanutbase.org/).

### Data analysis and filtering

For the detection of SNP and insertion/deletion (InDel) variants, we used GATK software (McKenna et al., 2010), and SnpEff software (Cingolani et al., 2012) was used to perform variant annotation and predict variant impact. In order to obtain high-quality SNPs for association analysis, the SNPs were initially filtered by removing SNP loci with multiple genotypes, and then those loci with read support values of less than 4. Parent SNP information was then used filter out those sites with different phenotypes derived from the same parent. The SNPs that remained were deemed credible.

### BSA-seq analysis

SNP-index is a marker association analysis method based on differences in genotype frequencies of mixed pools (Fekih et al., 2013). The main purpose of this method is to detect significant differences in the genotype frequencies of mixed pools using Δ (SNP-index) statistics (Fekih et al., 2013). The stronger the correlation between marker SNPs and traits, the closer Δ (SNP-index) is to 1. The Euclidean distance (ED) algorithm is a method where by sequencing data is used to detect significant differences between the markers of mixed pools and to evaluate the intervals associated with traits (Hill et al., 2013). In the context of the present study, with the exception of differences in sucrose content-related sites, other sites of the two mixed pools constructed using the BSA should be relatively consistent, and consequently, the ED values of non-target sites should be approximately 0. In contrast, the higher the ED value, the greater is the difference of the marker between the two mixed pools. In this study, we used *G* statistics for the purpose of gene detection. The *G* value of each SNP is calculated according to the allele sequencing depth, and is weighted according to the physical distance of the adjacent SNP (Magwene et al., 2011). In addition, given that *G* values are close to the lognormal distribution, the non-parametric estimation of the zero distribution of *G* values can be used to estimate the *p*-value of each SNP (Mansfeld and Grumet, 2018). When the values of *P* and the false discovery rate are both less than 0.01, it is considered that this interval may be the main effect area affecting the trait of interest (i.e., sucrose content in the present study).

Associations among the Δ (SNP-index), ED, *G* statistics, and *p* values of the SNP loci were analyzed using the website https://github.com/xiekunwhy/bsa. The four methods used are all based on a 2-Mb sliding window with a step size of 10 kb, which is applied to calculate the average and smooth the map. A 99% confidence level was selected as the threshold for screening, and the window above the confidence level was defined as the area associated with sucrose content. The intervals obtained using the four correlation analysis methods were compared, and the overlapping interval was regarded as the QTL interval associated sucrose content. The genes and polymorphic sites in the candidate interval were annotated using the website https://www.peanutbase.org/.

### Development of KASP markers and verification of the initial positioning results

Young leaves were collected from RIL population plants, and genomic DNAs were extracted using a plant genomic DNA extraction kit (DP305-03, TianGen). On the basis of the differential SNP information obtained for the two parents Jihuatian 1 and PI478819 in the initial mapping interval of the QTL, we designed 23 pairs of KASP primers using Primer Premier 5.0. FAM or HEX fluorescent splice sequences were attached to the 5′ ends of the primers and synthesized by the LGC Genomics company. The PCR reaction mixtures used contained the following: 1 μl of template DNA at a concentration of 50–100 ng/μl and 1 μl of a mixture of 1× Master Mix and Primer Mix. We performed LGC water bath PCR amplification, using the following amplification program: pre-denaturation at 94°C for 15 min; 10 cycles of denaturation at 94 °C and extension at 55°C–61°C for 1 min; 26 cycles of denaturation at 94°C and extension at 55°C for 1 min; and preservation at 10 °C. After the reactions were completed, the genotypes of each site were determined using the SNPline genotyping platform (Majeed et al., 2018).

QTLs for the sucrose contents in plants cultivated in each environment and at different stages of growth were detected based on the replication mean using QTL IciMapping (Li et al., 2007; Meng et al., 2015), setting the mapping step size as 1 cM and the logarithm of odds (LOD) threshold as 3.0. The QTL region of LG06 was drawn using MapChart 2.3 (Voorrips, 2002). QTLs were designated as follows: q+ the abbreviated trait name + linkage group number, or named as q+ the abbreviated trait name + linkage group number + a number designating one of multiple QTLs in a single linkage group, following the International Rules of Genetic Nomenclature (Liu et al., 2008).

### Candidate gene analysis

On the basis of BSA-seq analysis and fine mapping combined with gene annotation information, we performed a preliminary determination of candidate genes. Following a previously described procedure (Pattee et al., 1974), kernel tissue were collected from both parents at 20, 35, 50, and 60 days after flowering (stages S1–S4), with three biological replicates for each period. S1 is the early development stage, S2 and S3 are the developing stages, and S4 is the seed maturity stage. Total RNA was extracted from the collected tissues using a RNAprep Pure Plant Plus Kit (DP441, TIANGEN) and the concentration and purity of the extracted RNA were examined. High-quality RNA samples were selected based on the obtained purity values and concentration values were used to determine the amount of RNA template. The isolated RNA was subsequently reversed transcribed to cDNA using a FastKing RT Kit (With gDNase) (KR116, TIANGEN), and the cDNA thus obtained was diluted with sterile double-distilled water. qPCR reaction systems were prepared according to the requirements of a PowerUp SYBR Green Master Mix kit. A Quant Studio 5 real-time quantitative PCR instrument was used to run the reactions, and the 2^−ΔΔCT^ method was used to determine gene expression levels (Livak and Schmittgen, 2001). For each sample, we assessed three biological replicates, for each of which, we also analyzed three technical replicates. The relative expression of candidate genes at the different developmental stages of Jihuatian 1 and PI4788 was determined based on normalization analysis of the gene expression data, using the *ADH3* gene as an internal reference gene (Brand and Hovav, 2010). The cDNA sequences of candidate genes and *ADH3* were downloaded from the Peanutbase website (https://www.peanutbase.org/), and corresponding primers were designed using Primer Premier 5.0.

## Results

### Phenotypic identification of F_2_ and RIL populations

**FIGURE 1:**
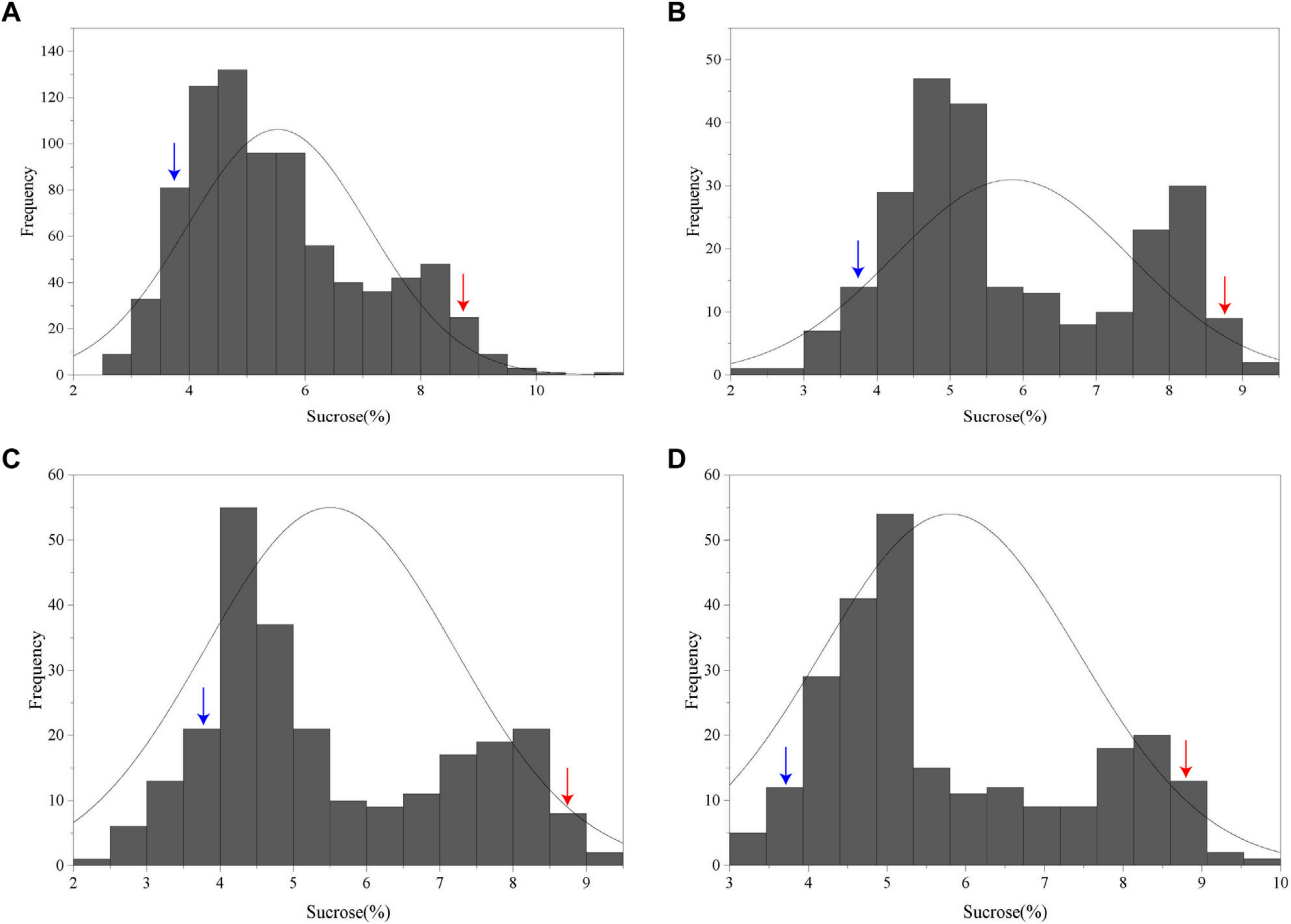
Frequency distribution of sucrose content in F_2_ and RIL populations of peanut (*Arachis hypogaea*). **(A)** Frequency distribution of sucrose contents in the F_2_ population. **(B–D)** Frequency distribution of sucrose content in the RIL population planted in Xinxiang, Kaifeng, and Zhumadian (Henan Province). The blue and red arrows indicate the sucrose contents of the male parent PI478819 and female parent Jihuatian 1, respectively.

NIR spectrometric analysis indicated that the sucrose contents of the female parent Jihuatian 1 and male parent PI478819 were 8.96% and 3.70%, respectively. For the sucrose content of the F_2_ population, we obtained maximum and minimum values of 11.03% and 2.52%, respectively, with a coefficient of variation of 0.28%, (Supplementary Table S1). The sucrose content per plant in the F_2_ population showed continuous variation and an approximate normal distribution, which is typical of a quantitative character (Figure 1A). The sucrose content of the RIL population was measured in three environments, with mean values of 5.89%, 5.64%, and 5.91% and coefficient of variation ranging from 0.29% to 032% being obtained (Table 1). In each of the three growth environments, we detected a continuous frequency distribution of sucrose content in the RIL population, indicating that the population may contain multiple major genes or QTLs associated with the control of sucrose content (Figures 1B–D). ANOVA revealed that sucrose content is influenced by genotype, the environment, and genotype–environment interactions (Table 2).

**TABLE 1:** Variation of sucrose content in different populations.

Population	Mean	SD	CV(%)	Min	Max	Kurt	Skew
F_2_	5.53	1.56	0.28	2.52	11.03	−0.29	0.68
F_9_-XX	5.89	1.68	0.29	2.26	9.34	−1.11	0.34
F_9_-KF	5.64	1.79	0.32	2.27	9.67	−0.92	0.45
F_9_-ZMD	5.91	1.69	0.29	2.78	9.92	−1.00	0.50

Note: F_2_: F_2_ segregation population; F_9_-XX: F_9_ RIL, population planted in Xinxiang; F_9_-KF: F_9_ RIL, population planted in Kaifeng; F_9_-ZMD: F_9_ RIL, population planted in Zhumadian. same as below.

**TABLE 2:** Analysis of variance for sucrose content in RIL population.

Sucrose	df	SS	MS	F-value	*p*-value
Genotype	250	3823.557	15.294	37.403	<0.01
Environment	2	34.744	17.372	42.484	<0.01
Genotype×Environment	500	262.067	0.524	1.282	<0.01
Error	753	307.905	0.409		

### Identification of candidate SNPs associated with sucrose content using BSA

Using the measured phenotype data, individuals with extreme phenotypes were used to form two mixed pools (Supplementary Table S1). The original data obtained from whole-genome re-sequencing of the two mixed pools and two parents were filtered to obtain a total of 336.63 Gbp clean reads, with a Q30 value ≥84.09%, GC content ranging from 36.66% to 38.26%, and the distribution of insert sizes showing a unimodal normal distribution. The average comparison efficiency between samples and the reference genome was 97.14%, the average sequencing depth was 32.92×, and we obtained 98.72% genome coverage (Table 3). The values of these parameters indicated the sufficiently good quality of sequencing and a high percentage matches with the peanut reference genome, thereby indicating that the obtained sequences could be used for subsequent variant detection and analysis.

**TABLE 3:** Sequencing data evaluation and comparison with reference genome statistics.

Sample_ID	Clean_reads	GC_rate (%)	Q20 (%)	Q30 (%)	Mapped (%)	Coverage_rate (%)	Mean_depth
JHT 1	439154736	38.26	92.55	84.09	95.54	98.53	25.76×
PI478819	604226914	37.47	95.69	89.88	96.81	98.87	35.45×
HSP	600610150	36.91	97.38	93.52	98.13	98.73	35.23×
LSP	600223092	36.66	97.11	92.93	98.07	98.74	35.21×

Note: Sample_ID, sample number; Clean_bases, number of bases filtered; Clean_reads, number of Clean reads filtered; GC (%), sample GC, content, that is, percentage of G and C type bases in total bases; Q20 (%), percentage of bases with mass value greater than or equal to 20 in total bases; Q30 (%), percentage of bases with mass value greater than or equal to 30 in total bases. Mapped (%), percentage of Clean Reads to reference genome in total Clean Reads; Coverage_ratio (%), percentage of overlay sites in genome; Mean—depth, average sequencing depth.

**FIGURE 2:**
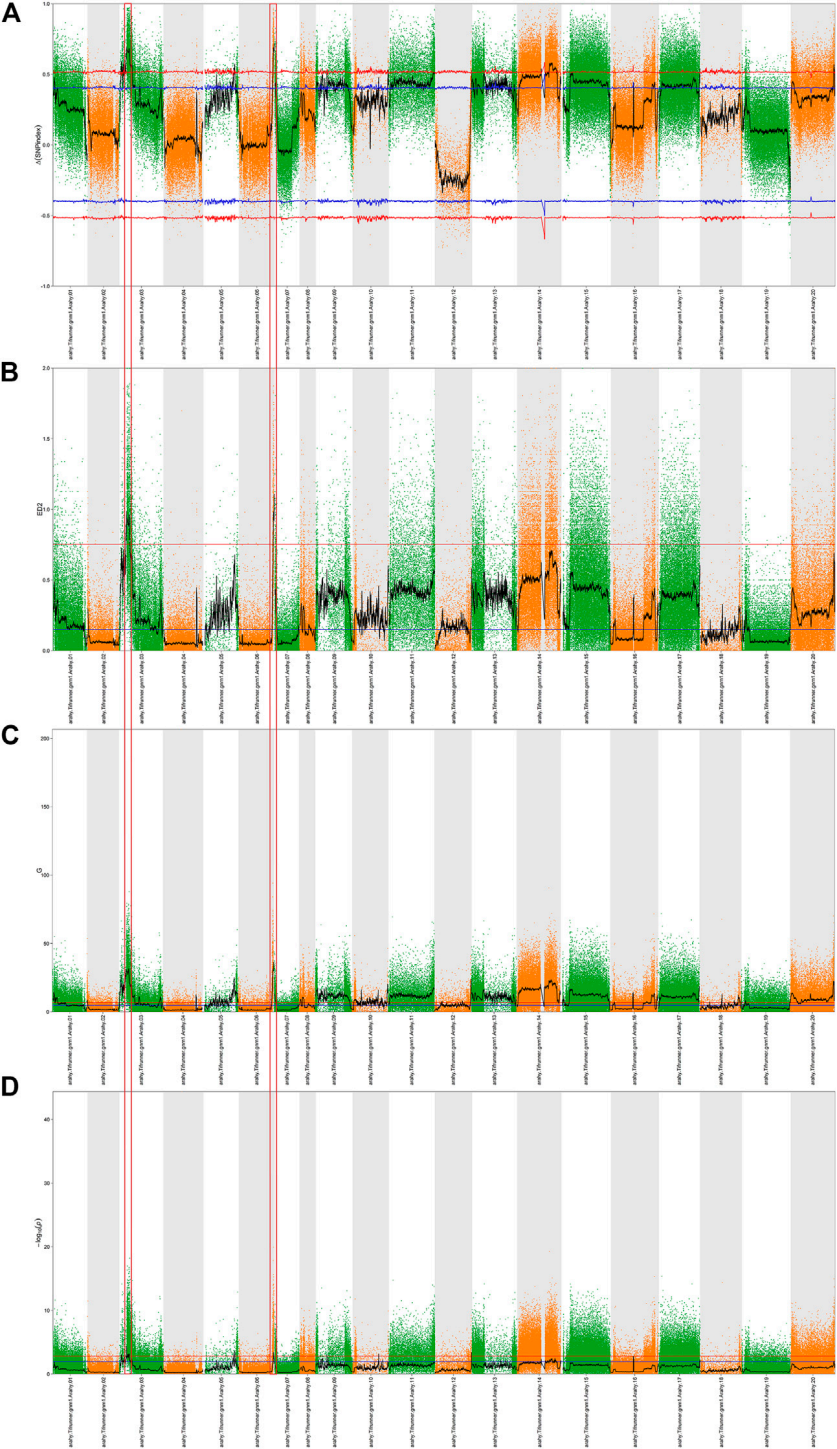
QTL analysis of sucrose based on BSA-seq. A Manhattan plot showing the distribution of Δ (SNP-index) **(A)**, the square of the Euclidean distance **(B)**, the distribution of *G*-values **(C)**, and the distribution of -log 10 (*p*-values) based on Fisher’s exact tests **(D)** on chromosomes. The blue and red lines represent 95% and 99% confidence intervals, respectively. The black lines are average values of the four algorithms and were drawn based on sliding window analysis. The numbers on the horizontal coordinates represent the chromosome numbers. The part circled by the red box is the target region.

Prior to bulked segregant analysis, we filtered out low-quality SNPs, and thereby finally obtained 318,057 high-quality credible SNPs. These high-quality SNPs were subjected to Δ (SNP-index) (Figure 2A), ED (Figure 2B), *G*-value (Figure 2C), and Fisher’s exact test (Figure 2D) association analyses, and plotted according to the chromosomal distribution of each parameter. Using a 99% confidence level as the screening threshold for associated chromosomal intervals, all four methods identified multiple candidate intervals on multiple chromosomes (Supplementary Table S2). The three overlapping intervals obtained using these four methods were identified as candidate intervals associated with peanut sucrose content (Table 4).

**TABLE 4:** Initial positioning QTL interval information.

Chromsome	Start(bp)	End(bp)	Size(Mb)
Arahy.03	5540001	8340000	2.80
Arahy.03	20800001	34180000	13.38
Arahy.06	109810001	114840000	5.03

### Verification and narrowing of the positioning range

**FIGURE 3:**
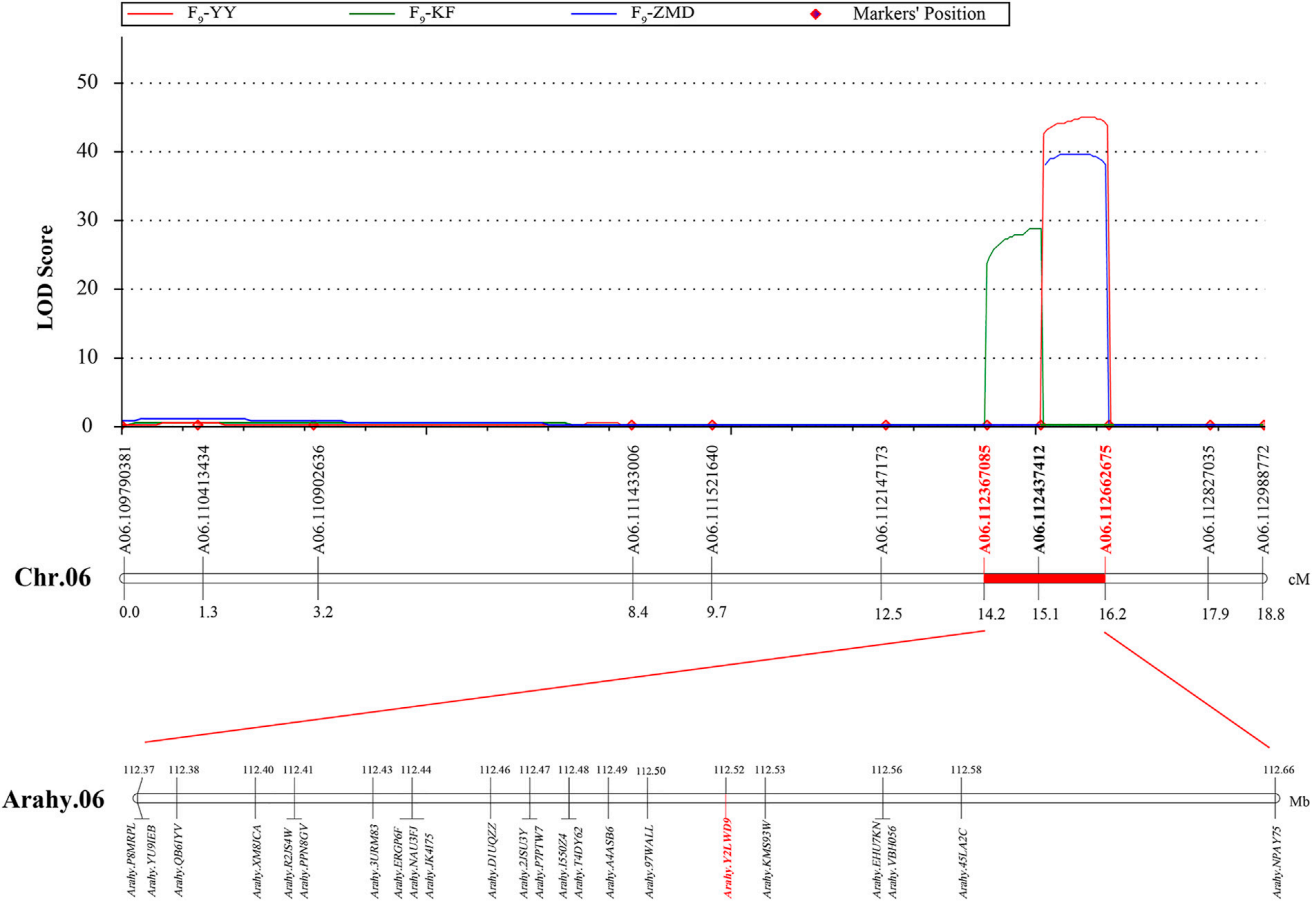
QTL fine mapping of sucrose content on peanut chromosome A06.

According to the different SNP information of Jihuatian 1 and PI478819 in three overlapping QTL intervals, the KASP primers were designed (Supplementary Table S3). The markers were genotyped in 251 RIL lines grown in three environments and subjected to genetic linkage analysis. The results revealed that the QTL detected in the initial mapping interval on chromosome A03 was not identified in the RIL population, indicating that this locus might be a false positive locus (Table 5). For all three assessed environments, we detected a candidate interval on chromosome A06 with phenotypic variance explained (PVE) and LOD values of 31.95%–41.05% and 28.70–44.84, respectively, which was considered to be a major QTL, which we designated *qSUCA06* (Figure 3). The genetic distance of the *qSUCA06* interval was 2.01 cM and the physical distance was 0.29 Mb (112367085–112662675 bp) (Table 5).

**TABLE 5:** QTL fine mapping of sucrose content in peanut kernels.

Environment	Chromsome	Position	LeftMarker	RightMarker	LOD	PVE(%)	Add
F_9_-XX	Arahy.06	15.70	A06.112437412	A06.112662675	44.84	41.05	−1.0196
F_9_-KF	Arahy.06	14.80	A06.112367085	A06.112437412	28.70	31.95	−0.9461
F_9_-ZMD	Arahy.06	15.20	A06.112437412	A06.112662675	39.61	37.64	−0.9842
F_9_-XX	Arahy.03	54.00	A03.31223142	A03.33012101	3.23	6.03	−0.3867
F_9_-KF	Arahy.03	13.00	A03.5557721	A03.6982931	2.58	4.68	−0.3863
F_9_-ZMD	Arahy.03	54.00	A03.31223142	A03.33012101	2.90	5.75	−0.3721

### Candidate gene annotation and expression analysis

The total of 23 genes were identified in the *qSUCA06* interval (Figure 3, Supplementary Table S4). And then we detected eight genes changed in exon regions by further amplification and identification in this interval. Among which, six and three genes characterized by SNP and InDel differences, respectively, between Jihuatian 1 and PI478819 (Supplementary Table S5). We have made in-depth functional annotation on several databases and identified two genes related to protein synthesis and metabolism, three genes related to signal transduction, two genes related to cell cycle, and one gene encoding transcription factor through the analysis of the biological process of gene expression products.

**FIGURE 4:**
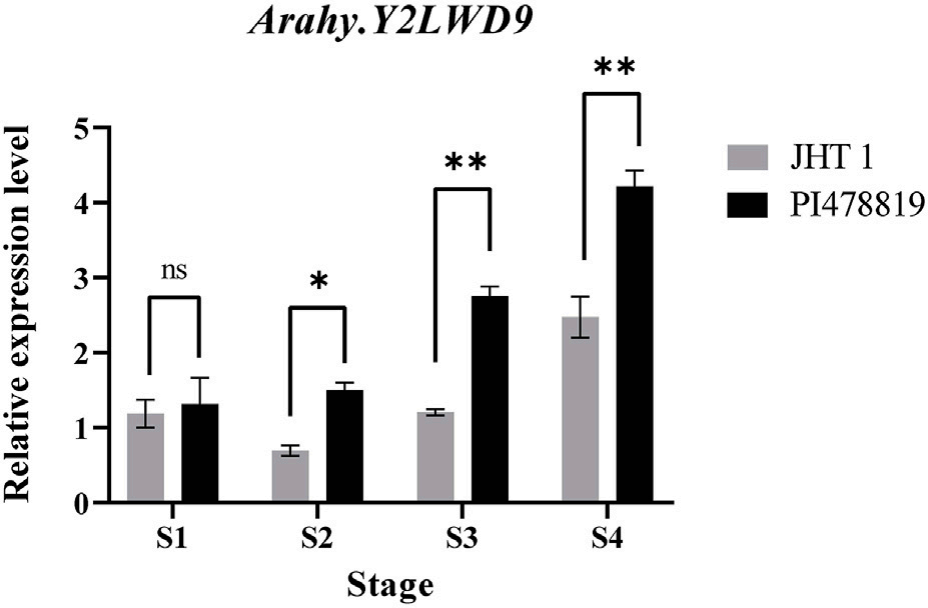
Expression analysis of a candidate gene in two parents at different growth stages (S1 to S4). Asterisks indicate a significant difference between the Jihuatian 1 (JHT 1) and PI478819 parents at each of the four growth stages, as determined using Student’s *t*-test (^ns^P > 0.05; *p < 0.05; **p < 0.01).

The candidate gene designated *Arahy.Y2LWD9*, which encodes acyl-CoA-binding domain 3 (ACBD), is a domain of acyl-CoA-binding proteins, a class of lipid transporter family proteins, which may be associated with sucrose. Given the detected correlation between *Arahy.Y2LWD9* and sucrose accumulation, we analyzed the levels of *Arahy.Y2LWD9* expression in the two parents. On the basis of the cDNA sequences of *Arahy.Y2LWD9*, we designed primers (Table 6) and performed qRT-PCR analyses of candidate genes, using *ADH3* as the internal reference control (Brand and Hovav, 2010). The results showed that whereas there were no significant difference between two parents at the S1 stage of seed development with respect to the relative expression of *Arahy.Y2LWD9*, we detected significant differences in expression at stages S2, S3, and S4 (Figure 4). Overall, the expression of *Arahy.Y2LWD9* in the two parents showed an upward trend, which was opposite to the observed accumulation of sucrose, thereby tending to indicate this gene may play a negative regulatory role in the accumulation of sucrose in peanut (Li et al., 2021).

**TABLE 6:** The information of primers for quantitation real-time PCR.

Gene ID	Forward primer	Reverse primer	Product Length(bp)
*Arahy.Y2LWD9*	ATG​AAC​CTC​AAC​CAA​TGC​CTC​T	CAG​GAA​CAG​CAA​ACC​CAG​AA	204
*ADH3*	GAC​GCT​TGG​CGA​GAT​CAA​CA	AAC​CGG​ACA​ACC​ACC​ACA​TG	140

## Discussion

Given their high nutritional value, peanuts are probably the most widely consumed type of nut. For consumers, it is desirable that peanut kernels are of high quality with a good taste, an important contributory factor of which is sucrose content, which imparts a sweet taste. In this study, using BSA-seq technology, we investigated the potential genetic mechanisms underlying the control of sucrose content of peanut. For the purposes of QTL mapping analysis, we used an F_2_ segregating population of 831 plants and an RIL population comprising 251 lines. Our ANOVA results revealed that the growth environment has a significant influence on the sucrose content of peanut kernels. To minimize the effect of environment on the mapping results, we cultivated the RIL population in three different locations, which also contributed to a more accurate and reliable identification of QTLs.

To date, there have been a few studies that have examined the QTLs or genes associated with sucrose content in peanut kernels. In one of these studies, the transcriptomes of two peanut cultivars with different sucrose contents were comparatively analyzed based on weighted gene correlation network analysis and qRT-PCR across multiple developmental stages, and six genes with high expression levels were finally identified in the derived RILs (Li et al., 2021). However, whereas none of the six genes reported were detected within the confidence intervals of the QTL, the QTL *qSUCA06* identified in present study was found to have a negative additive effect with a PVE of 31.95%–41.05%, which accordingly tended to indicate the high probability that novel genes regulating sucrose content are located in this region.

The candidate gene *Arahy.Y2LWD9* identified within the *qSUCA06* QTL, which encodes acyl-CoA-binding domain 3. might be one of such gene modulating sucrose content. Previous studies have shown that ACBD is a domain of acyl-CoA-binding proteins, which play key roles in plant fat metabolism (Ye and Chye, 2016). In eukaryotic cells, these proteins are involved in the transport of acyl-CoA esters and the formation and maintenance of the cytosolic acyl-CoA pool, thereby contributing to the regulation of lipid metabolism (Guo et al., 2017). On the basis of principal component analysis, Yu et al. (2020) found that the sucrose content in peanut kernels was negatively correlated with fat content. In the present study, we detected significant differences between the two parents with respect to the expression of *Arahy.Y2LWD9* during different stages of development, and that overall, there was an upward trend in expression with growth progression, which was opposite to the accumulation of sucrose. Acyl-CoA-binding proteins contain a class of highly conserved acyl coenzyme A that has been identified from rice (Meng et al., 2011), *Arabidopsis thaliana* (Xiao and Chye, 2009), *Agave americana* (Guerrero et al., 2006), and *Brassica napus* (Hills et al., 1994). Studies have shown that overexpression of *OsACBP2* in rice can promote a significant increase in the contents of triglycerides and long-chain fatty acids in seeds (Guo et al., 2019). In view of the strong activity of *Arabidopsis thaliana AtACBP6 pro::GUS* in the cotyledons of developmental embryos and the accumulation of oleyl and linoleyl CoA esters in ACBP6 seedlings, it is speculated that *AtACBP6*, together with *AtACBP4* and *AtACBP5*, may play a role in seed oil synthesis (Hsiao et al., 2014). Consequently, it is plausible that *Arahy.Y2LWD9* indirectly regulates sucrose content by regulating lipid metabolism in peanut kernels; however, this specific function needs to be further verified based on either overexpression or loss-of-function analyses.

## Conclusion

4In this study, we identified a major stable QTL, *qSUCA06*, for peanut sucrose content based on BSA-seq analysis and fine mapping. Within the QTL interval, we detected a candidate gene, *Arahy.Y2LWD9*, which was verified by qRT-PCR to be negatively corrected with peanut sucrose content. The findings of this study provide a theoretical basis for further analysis of the genetic regulation of sucrose content in peanut, and will contribute to breeding for both oil and sucrose contents, taking into consideration the requirements of industry and consumers.

## Data Availability

The datasets presented in this study can be found in online repositories. The names of the repository/repositories and accession number(s) can be found below: https://bigd.big.ac.cn/gsa/browse/CRA009024.

## Abbreviations

AFLP, amplified fragment length polymorphism; ANOVA, analysis of variance; ACBP, acyl-CoA-binding protein; BSA, bulk segregation analysis; ED, Euclidean distance; GATK, The genome analysis toolkit; KASP, Kompetitive allele-specific PCR; LG, linkage group; LOD, log of odds (genetic linkage score); NIR, near infrared; PVE, phenotypic variation explained; QTL, quantitative trait locus; RIL, recombinant inbred line; qRT-PCR, quantitative real-time PCR; RFLP, restriction fragment length polymorphism.
